# Reliable delineation of *Clostridioides difficile* and related members of the family Peptostreptococcaceae using phylogenomics and spore coat protein-specific molecular markers

**DOI:** 10.1128/spectrum.04185-25

**Published:** 2026-06-15

**Authors:** Jianying Han, Yannan Li, Yuxi Xu, Shaoting Li, Ji Zeng

**Affiliations:** 1 The School of Biomedical and Pharmaceutical Sciences, Guangdong University of Technology 47870, Guangzhou, China; Newcastle University, Newcastle Upon Tyne, United Kingdom

**Keywords:** Peptostreptococcaceae, *Clostridioides difficile*, delineation, spore coat protein

## Abstract

Traditional bacterial classification relies on phenotypic traits (e.g., morphology and metabolic profiles), but these methods lack resolution for closely related taxa and are biased by culture conditions. While 16S rRNA gene sequencing is a widely used molecular complement, it fails to resolve closely related Peptostreptococcaceae species, including *Clostridioides difficile*. These limitations have caused family-level taxonomic confusion and ambiguous *Clostridioides* genus boundaries, hindering clinical identification of pathogenic strains and posing public health risks. To address these limitations, we developed an integrated approach combining multi-scale phylogenomic and protein-based molecular evidence, adopting a hierarchical workflow: first, constructing a 16S rRNA phylogeny of 151 *Firmicutes* strains to demonstrate traditional marker inadequacies; second, generating a whole-genome protein phylogeny of 51 representative Peptostreptococcaceae genomes and defining taxonomic boundaries via average amino acid identity (AAI); third, analyzing spore-associated protein patterns across *C. difficile* isolates and related genomes. Results revealed high conservation of *C. difficile* spore coat/exosporium proteins and clear genus-level phylogenetic distinctiveness of these proteins. Combined with AAI-validated whole-genome data, our findings support key Peptostreptococcaceae taxonomic revisions: redefining polyphyletic *Romboutsia*, reassigning *Eubacterium tenue* to *Paraclostridium*, and elevating *Alkalithermobacter* to genus status. This study establishes spore coat proteins as core taxonomic markers for spore-forming bacteria, with our integrated strategy overcoming traditional limitations to improve classification accuracy and *C. difficile* surveillance.

**IMPORTANCE:**

Conventional classification struggles to resolve closely related Peptostreptococcaceae species (e.g., *Clostridioides difficile*). We developed an integrated framework combining 16S rRNA sequencing, whole-genome protein analysis, and spore trait assessment, with a key innovation: identifying spore coat/exosporium proteins as robust, conserved taxonomic markers. This approach enabled three pivotal Peptostreptococcaceae revisions—redefining Romboutsia, reassigning *Eubacterium tenue* to *Paraclostridium*, and elevating Alkalithermobacter to genus rank. The findings resolve a longstanding microbial systematics bottleneck for spore-forming bacteria, provide critical taxonomic context for *C. difficile*’s precise monitoring and prevention, and expand taxonomic markers beyond nucleic acid-based methods. This advances classification precision, critical for microbial ecology, pathogenesis, and industrial microbiology research.

## INTRODUCTION

Accurate classification of bacterial species and genera is fundamental to microbiological research, enabling critical insights into microbial ecology and function (1), with direct implications for human health (2). Nowhere is this more evident than in clinical medicine (2), where precise taxonomic assignment of pathogens is indispensable for elucidating mechanisms of virulence and developing targeted interventions (3). This necessity is starkly illustrated by *Clostridioides difficile*, a leading cause of antibiotic-associated diarrhea globally. *C. difficile* infection presents a spectrum of disease, from mild diarrhea to life-threatening pseudomembranous colitis, and is notably prone to recurrence (4), incurring healthcare costs amounting to billions of dollars annually (5, 6). Concerns are amplified in regions like China, where high toxin detection rates in tertiary hospitals suggest a potentially underestimated epidemic burden (7, 8). These clinical realities underscore the imperative to resolve the precise phylogenetic position and taxonomic boundaries of *C. difficile*.

The taxonomic home of *C. difficile* lies within the family Peptostreptococcaceae, a group whose classification requires rigorous phylogenetic analysis. *C. difficile* itself was reclassified from the genus *Clostridium* to *Clostridioides* following genomic evidence (9). Peptostreptococcaceae species, which inhabit diverse niches including the human gut and soil, often share key biological traits with *C. difficile*, such as anaerobic metabolism and sporulation (10). However, the current taxonomy of this family is fraught with challenges that obscure phylogenetic relationships (11). Traditional phenotypic methods lack the resolution to distinguish between closely related genera that share these common traits (12), and their results can be biased by culture conditions, ultimately compromising the accuracy of taxonomic assignments and leaving the placement of many strains ambiguous (10, 13).

While molecular sequencing promised to overcome these limitations, both 16S rRNA and whole-genome approaches exhibit specific shortcomings that perpetuate taxonomic controversies (14). The 16S rRNA gene, a cornerstone of bacterial taxonomy, is too conserved for reliable species-level discrimination. Within Firmicutes, this is exemplified by near-identical 16S sequences (>99.5% similarity) between *Bacillus subtilis* and *Bacillus amyloliquefaciens* (15) and minimal differences between *C. difficile* and close relatives like *Clostridium sordellii* (16). Whole-genome sequencing, though more powerful, provides a definitive resolution in such cases, as demonstrated by its role in underpinning the reclassification of *C. difficile*, but it introduces challenges of cost, analytical complexity, and the difficulty of extracting phylogenetically robust signals from massive data sets (17).

These technical limitations are concretely reflected in the persistent taxonomic ambiguities within Peptostreptococcaceae. A critical issue is the non-monophyly of some described genera (12). The genus *Romboutsia*, for example, is not a natural group; some of its species show a closer phylogenetic affinity to *Paraclostridium* than to other *Romboutsia* members, blurring the evolutionary picture around *C. difficile* (10). Similarly, the species *Eubacterium tenue* displays ambiguous clustering in phylogenetic trees, sometimes associating with *Paraclostridium* and other times with *Clostridioides*, which hinders a clear understanding of its relationship to *C. difficile*. These specific cases underscore that accurate phylogenetic reconstruction of the entire family remains an unresolved challenge, one that directly impedes the clarification of *C. difficile*’s closest relatives.

Given the constraints of sequence-based methods, functional biomarkers offer a compelling complementary strategy for taxonomic delineation. While toxinotype classification reliably differentiates virulent lineages within *C. difficile*, these markers reflect pathogenic variation among closely related strains rather than deeper phylogenetic divergence between species and genera across the Peptostreptococcaceae. Unlike toxins, core spore coat proteins address this critical gap. Although basic spore structural frameworks are broadly conserved across spore-forming Firmicutes (18), the primary sequence divergence of key spore coat proteins (e.g., SpoIVA, YabG, and CotE) is highly specific to distinct phylogenetic lineages. Because these proteins evolve under tight functional constraints for sporulation and germination, they yield a stable phylogenetic signal that distinguishes genera and species instead of merely reflecting strain-level virulence. Critically, our comparative analysis confirms that such species- and genus-specific conservation is pronounced within Peptostreptococcaceae. Core spore coat proteins maintain high intra-species identity while accumulating diagnostic inter-species differences; furthermore, many lack detectable homologs in distantly related spore-formers such as *Bacillus subtilis*. Consequently, these proteins are uniquely suitable to resolve longstanding phylogenetic ambiguities in the family, where 16S rRNA and whole-genome signals remain limiting. Despite this potential, a comprehensive taxonomic framework based on spore coat proteins for Peptostreptococcaceae remains to be established, and its power to resolve phylogenetic disputes requires systematic validation.

To address these gaps, our study employs an integrated strategy that combines robust genomic phylogeny with a targeted analysis of these lineage-specific spore protein markers. We aim to resolve the taxonomic ambiguities within the Peptostreptococcaceae family, thereby clarifying the phylogenetic context of *C. difficile* and refining the boundaries of the genus *Clostridioides*. Our methodology proceeds in three concerted steps: first, to establish a robust phylogenetic framework using 16S rRNA and whole-genome sequences; second, to investigate the phylogeny of *C. difficile* spore coat proteins through systematic analysis of their conservation, diversity, and homology; and finally, to validate the resulting classification model using average amino acid identity (AAI). This multi-faceted approach is designed to yield a reliable taxonomy that will support future ecological studies and the precise clinical identification of pathogens within this critical family.

## MATERIALS AND METHODS

### Genomic data collection and preprocessing

First, genomic sequences related to the family Peptostreptococcaceae were retrieved from the public genomic database NCBI RefSeq (19). To ensure the representativeness of this study, 51 complete genomes were downloaded. Among these, two genomes corresponded to *Faecalimicrobium dakarense* FF1 and *Maledivibacter halophilus* M1, which were used as outgroups for constructing the whole-genome phylogenetic tree. The remaining 49 genomes included various bacteria isolated from human or animal tissues (e.g., intestine/feces, oral cavity, and blood) and represented multiple genera, namely *Acetoanaerobium*, *Alkalithermobacter*, *Asaccharospora*, *Clostridioides*, *Clostridium*, *Criibacterium*, *Eubacterium*, *Filifactor*, *Intestinibacter*, *Lachnospira*, *Mediannikoviicoccus*, *Metaclostridioides*, *Paeniclostridium*, *Paraclostridium*, *Paramaledivibacter*, *Peptacetobacter*, *Peptoanaerobacter*, *Peptoclostridium*, *Peptostreptococcus*, *Proteocatella*, *Romboutsia*, *Tepidibacter*, *Terrisporobacter*, and *Wukongibacter*.

The selection criteria for the genomic sequences were as follows: (i) all sequences were complete genomes; (ii) the sources covered multiple host types (e.g., humans and animals) to fully reflect the diversity of the Peptostreptococcaceae family across different ecological environments; (iii) all genomes had clear gene annotations and functional annotations, ensuring the reliability of subsequent analyses. All 51 genomes were complete, pre-annotated RefSeq assemblies, and their annotated protein sequences were directly used for ortholog identification and phylogenetic analysis.

Additionally, genomic data of *C. difficile* were obtained from the NCBI SRA database (20). In this study, 280 genomic sequences of *C. difficile* strains isolated from the United States were retrieved, covering 23 distinct PCR ribotypes from both clinical and environmental origins. These PCR ribotypes included PCR ribotype 027 (a hypervirulent epidemic strain of *C. difficile*), PCR ribotype 078 (a zoonosis-related strain), PCR ribotype 106, and PCR ribotype 147. Detailed information regarding the accession numbers, origins, and relevant metadata for these 280 *C. difficile* genomes is provided in Table S1. These data were primarily used for systematic analysis of the presence, sequence conservation variations, and genomic distribution characteristics of key sporulation genes (e.g., *spoIVA*, *cotE*, and *sleC*) across *C. difficile* strains. Moreover, they provided basic data support for subsequent studies on the associations among sporulation ability, strain virulence, and environmental adaptability.

The raw sequencing data (fastq format) of the 280 *C. difficile* strains in this study underwent comprehensive quality control and *de novo* assembly. The specific steps and tools are detailed below. First, raw sequencing data quality assessment was performed using FastQC v0.11.9 (21). Subsequently, low-quality sequences from the raw paired-end Illumina reads (150 bp read length) were filtered using Trimmomatic v0.39 (22), with a sliding window quality threshold of Q20 and a minimum read length of 50 bp. The quality-controlled reads were subjected to *de novo* assembly using SPAdes v3.15.3 (23). Following assembly, redundant sequences were further cleaned using CD-HIT v4.8.1 (24) with a sequence identity threshold of 95% (-c 0.95) and coverage thresholds of 90% for both longer (-aL 0.9) and shorter (-aS 0.9) sequences, in order to eliminate duplicate and highly similar sequences.

Finally, gene and protein annotation for these assembled sequences was conducted using Prokka v1.14.6 (25). Default parameters were supplemented with manual correction to focus on extracting structural and functional information of spore formation-related genes, ensuring the reliability of subsequent analyses.

### Analysis of 16S rRNA gene sequences

To construct a 16S rRNA-based phylogenetic tree and validate the taxonomic rationality of strains within the phylum Firmicutes, this study first established the screening criteria and sources for 16S rRNA gene sequences. A total of 151 bacterial 16S rRNA gene sequences were downloaded from the NCBI GenBank database (26), selected based on multiple peer-reviewed studies and stringent quality filters. These sequences met the following 16S rRNA-specific quality requirements: sequence integrity ≥95%, base call error rate <0.1%, and annotation completeness ≥90%. Importantly, while the Firmicutes phylum comprises substantially more than 151 strains, the final 151 sequences included in this analysis were determined by integrating literature-derived evidence with the aforementioned quality benchmarks. Additionally, the corresponding strains were sourced from typical Firmicutes niches (e.g., human gut, animal gut, soil, water, and clinical specimens) to ensure representative sampling.

The 151 sequences corresponded to strains belonging to 16 identified families, 1 unidentified family, and 48 genera, covering most known representative families and genera within Firmicutes. The 16 identified families included Peptostreptococcaceae, Bacillaceae, Alicyclobacillaceae, Peptoniphilaceae, Lachnospiraceae, Paenibacillaceae, Clostridiaceae, Cytobacillaceae, Enterococcaceae, Eubacteriaceae, Atopobiaceae, Neomoorellaceae, Tepidibacteraceae, Streptococcaceae, Thermoanaerobacteraceae, and Sporolactobacillaceae. The unidentified family was temporarily classified as an unclassified family of Firmicutes. The 48 genera included *Clostridioides* and *Paraclostridium* (from Peptostreptococcaceae), *Bacillus* and *Paenibacillus* (from *Bacillaceae*), and *Lachnospira* and *Roseburia* (from *Lachnospiraceae*), among others (Table S2).

Maximum likelihood (ML) phylogenetic analysis was conducted following a standardized pipeline. First, multiple sequence alignment (MSA) of the 16S rRNA gene sequences was generated using MAFFT v7.490 (27) with the G-INS-i algorithm, which is well suited for aligning highly conserved gene sequences such as 16S rRNA due to its accuracy in resolving positional homology. The optimal nucleotide substitution model was then determined using ModelFinder (28) as implemented in IQ-TREE 2 (29), with the TVMe + R10 model selected as best-fitting under the Bayesian Information Criterion. Phylogenetic tree reconstruction was subsequently performed using IQ-TREE 2 with 1,000 bootstrap replicates to evaluate branch support. Finally, the resulting tree was visualized and annotated using FigTree v1.4.4.

### Construction of a phylogenetic tree based on strain protein sequences

Using the protein sequences of 51 Peptostreptococcaceae strains, detailed in Table S3, conserved proteins (single-copy orthologous proteins) were first screened using OrthoFinder v2.5.4 (30) combined with Diamond v2.1.8 (31, 32). Specifically, OrthoFinder generated orthologous gene clusters via an orthology inference workflow based on gene clustering, while Diamond performed sequence alignment using the Smith–Waterman algorithm (--more-sensitive mode, *E*-value = 1e-10). The screening of protein sequences strictly followed four criteria: (i) each strain contained only one sequence (strict single copy); (ii) all 49 strains were covered; (iii) the coefficient of variation of sequence length was ≤20% (additionally, abnormal sequences with length <100 aa or >1,000 aa were excluded); (iv) average amino acid identity (AAI) ≥70%. The final set of single-copy orthologs used for phylogenetic tree construction is listed in Table S4.

Subsequently, MUSCLE v5.1 (33) was employed for multiple sequence alignment of each screened single-copy orthologous protein individually, with multi-round iterative optimization to improve alignment accuracy in conserved regions. Based on the MSA results, Gblocks v0.91b (34) was used for trimming, whereby only conserved blocks of length ≥5 aa were retained, semi-conserved gaps were allowed (with a maximum gap ratio ≤50%), and fragments containing >8 consecutive non-conserved sites or gap length >10 amino acids were removed. After trimming, SeqKit v2.4.0 (35) was used to concatenate sequences by strain, by connecting the trimmed sequences of all single-copy genes of each strain end-to-end in the order of gene IDs to form a continuous long sequence. Finally, IQ-TREE v2.2.6 (29) was used to construct the phylogenetic tree; this tool automatically selected the optimal amino acid substitution model and evaluated statistical branch reliability via 1,000 ultra-fast Bootstrap replicates and aLRT tests (where Bootstrap values ≥70% were defined as indicating reliable branches). Subsequently, tree visualization and annotation were performed using the web-based tool tvBOT (36).

### Conservation of spore coat proteins

Genomic data from 280 U.S.-isolated *C. difficile* strains were collected, see Table S3. Concurrently, 54 spore coat proteins and 21 exosporium proteins were screened from the genome-wide proteome of *C. difficile* type strain 630 (GenBank: NC_009089.1), and their conservation was analyzed.

First, BLASTp was used for homologous sequence searches against all 280 strains’ proteomes. Hits were defined as conserved spore-related proteins only if they met simultaneous criteria: Bit score ≥ 100, *E*-value < 1e-10, sequence identity ≥ 90%, and sequence coverage ≥ 80%.

Subsequently, the conservation degree distribution of each spore-related protein across the 280 genomes was analyzed. Here, conservation degree refers specifically to the frequency of a spore-related protein being detected as conserved (i.e., BLASTp-identified homologs) among the 280 strains. This analysis followed three steps: first, counting strains with each conserved spore-related protein; second, calculating detection rates as (positive strains / 280) × 100%; third, classifying into grades (e.g., highly conserved: ≥80% detection; moderately conserved: 50%–80%; poorly conserved: <50%) to visualize prevalence differences across proteins and strains.

### Multiple sequence alignment and phylogenetic analysis of spore coat proteins

Local BLASTp searches were performed against the proteome sequences of 49 Peptostreptococcaceae strains, using 54 *C. difficile* spore coat protein sequences and 21 *C. difficile* exosporium proteins as queries. An *E*-value threshold of 1e-2 was set to filter random matches, and a sequence identity threshold of ≥60% was used to ensure homology reliability. High-confidence homologous protein matches were obtained via these screening criteria.

Based on the screening results, a Presence/Absence matrix was constructed. If a strain’s protein sequence aligned with a spore coat protein and met the *E*-value and identity criteria, the corresponding position in the matrix was marked as “1” (present); otherwise, it was marked as “0” (absent). The presence/absence matrix was used to construct a neighbor-joining (NJ) tree in MEGA X (37) with 1,000 bootstrap replicates for reliability evaluation. The Jaccard coefficient was used to calculate the distance between matrix elements, ultimately generating a phylogenetic topology with confidence support.

All analyses were performed in R version 4.4.2 (38). We assessed protein distribution by generating a heatmap with ggplot2 (39) to visualize the presence of 54 spore coat proteins and 21 exosporium proteins across 49 strains, following data organization with the tidyverse suite (40). Phylogenetic trees were constructed and manipulated separately using ape (41) and treeio (42) and visualized with ggtree. All graphics were finalized using color schemes from RColorBrewer and assembled with patchwork.

### Multiple sequence alignment and phylogenetic analysis of core spore coat proteins

Based on the presence/absence matrix of 54 spore coat proteins and 21 exosporium proteins, a strict screening process identified three core spore coat proteins stably present in all 49 strains. MAFFT v7.490 (27) was used to perform multiple sequence alignment on these three core proteins. According to protein characteristics, trimAl v1.4.rev15 (43) was used to remove extensive “-” (insertions/deletions) from both ends of the sequences and retain core conserved regions, yielding high-quality alignment results. The final alignment results were carefully checked and optimized using the Jalview visualization tool (44).

Based on the MSA results of the three core spore coat proteins, a standardized amino acid variation matrix was constructed. RAxML-NG software (45) was used to construct the phylogenetic tree and evaluate its reliability. Using the LG + G + F substitution model (46), which accounts for amino acid frequencies and rate heterogeneity across four GAMMA categories (47), an ML phylogenetic tree was constructed with 1,000 bootstrap replicates. Finally, FigTree was used for tree visualization to present interspecific genetic relationships and branch reliability.

### Calculation of AAI

To evaluate the genetic relationships among 49 genomes, the CompareM toolkit (48) was used to calculate the AAI of conserved proteins across the whole genome. First, the Prodigal tool (49) was used to predict coding genes in each genome. Protein sequences with a length ≥100 aa were then screened and extracted. Pairwise sequence alignment was performed using BLASTp (*E*-value ≤ 1e-5), and orthologous gene pairs were screened using the following criteria: alignment coverage ≥70% and similarity ≥30%. The amino acid identity of each gene pair (AAI*ᵢ*) was calculated, and the average value was taken as the final AAI (formula: AAI = (1/*n*)∑*ⁿᵢ* ₌ ₁ AAI*ᵢ*, where *n* is the total number of orthologous gene pairs). The judgment criteria were as follows: AAI ≥ 95% (50) indicated closely related taxa; AAI ≤ 70% (51) indicated distantly related taxa (potentially belonging to different genera).

### Construction of the spore coat gene co-expression network

Gene expression data were obtained from the STRING database (52). A set of 54 spore coat genes and 21 exosporium-associated genes was initially queried to retrieve complete expression profiles under multiple sporulation-related conditions, including different sporulation stages, nutrient deprivation, and environmental stress. Owing to limitations in the database’s gene annotation coverage, which primarily stems from the inability to detect a subset of genes in the available experimental systems, presumably because of low expression levels, only 27 genes were successfully matched with valid expression data. Subsequent co-expression network analysis was therefore restricted to these 27 genes. The network was visualized using Cytoscape v3.7.2 (53), which clearly delineated co-expression relationships among these spore coat genes and provided an intuitive model for subsequent investigation into the molecular regulatory mechanisms underlying spore coat assembly.

## RESULTS

### Phylogenetic analysis of the 16S rRNA gene: preliminary insights into the polyphyly of the peptostreptococcaceae family

**Fig 1:**
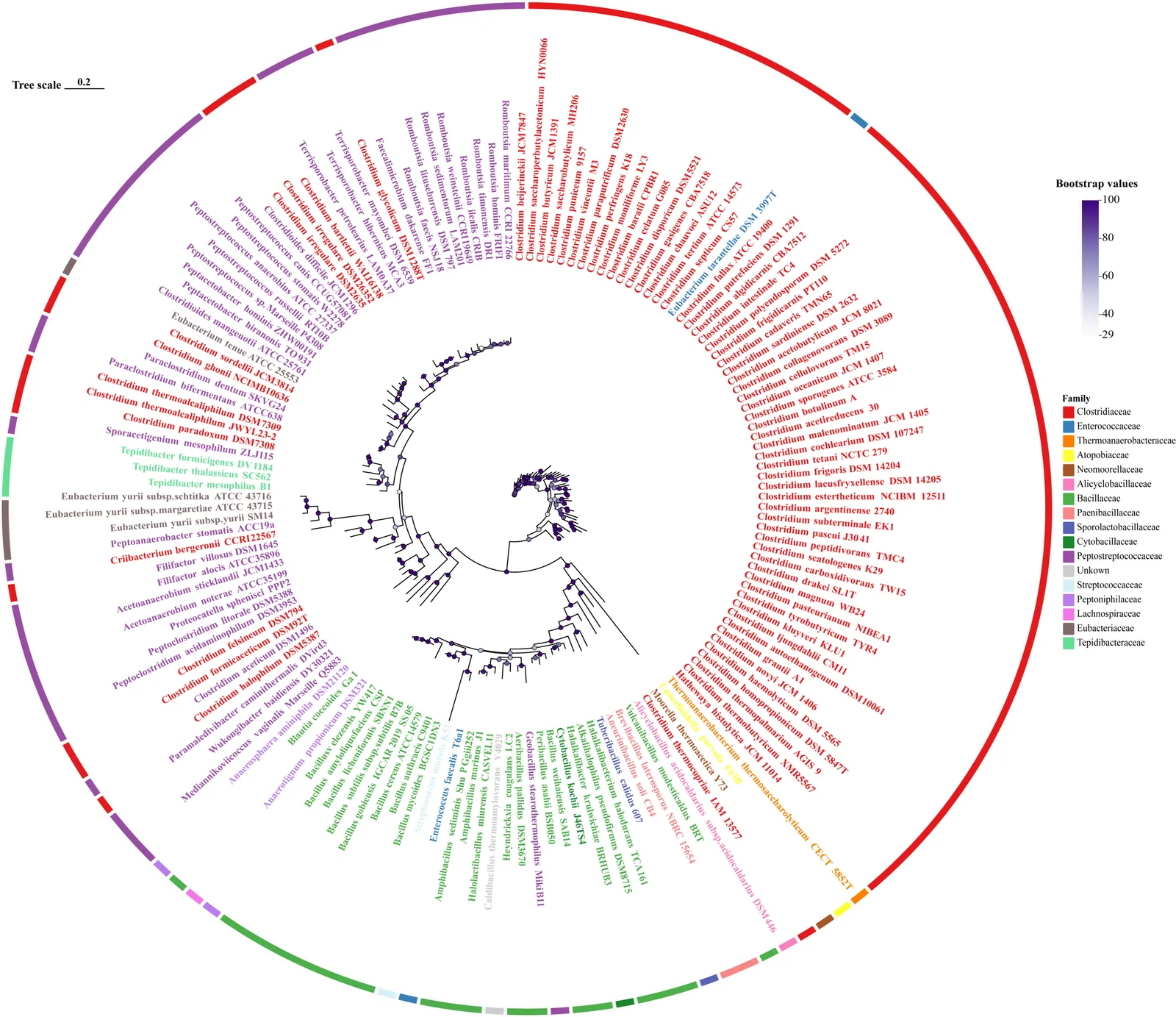
Maximum likelihood phylogenetic tree based on 16S rRNA gene sequences. Circular phylogenetic tree of 151 strains within the phylum Firmicutes, constructed using 16S rRNA gene sequences. The tree was generated via the maximum likelihood method, with values at nodes representing bootstrap support values (1,000 replicates).

To broaden the analysis of overall evolutionary relationships within the phylum *Firmicutes* and verify the universality of taxonomic controversies within the *Peptostreptococcaceae*, this study first reconstructed a phylogenetic tree using 16S rRNA gene sequences from 151 representative strains (Fig. 1). These strains encompass multiple genera and species within the *Peptostreptococcaceae*, with taxonomic annotations strictly referenced from the List of Prokaryotic Names with Standing in Nomenclature (54) or the Genome Taxonomy Database (55). This analysis provides a broad phylum-level perspective, highlighting the limited resolution and paraphyletic signals associated with the single-gene marker 16S rRNA.

Phylogenetic analysis based on 16S rRNA gene sequences revealed that the *Peptostreptococcaceae* family, as defined under the current taxonomic system, is a highly paraphyletic group. Its members do not cluster into a single evolutionary clade but are scattered across at least four major independent lineages, structurally confirming the lack of monophyly within this family. Specifically, the clade containing *Terrisporobacter petrolearius* and *Terrisporobacter mayombei* formed a tight subclade (98%–99% bootstrap support), which was closely intertwined with *Clostridium glycolicum* and *Clostridium felsineum* (*Clostridium* species) with 100% nodal support, thereby disrupting the monophyletic boundary of the *Peptostreptococcaceae*. Furthermore, although *Romboutsia* species (including *Romboutsia faecis*, *Romboutsia hominis*, and *Romboutsia ilealis*) formed a distinct genus-level clade (77%–88% bootstrap support), this clade still shows close phylogenetic affinities with non-Peptostreptococcaceae species, suggesting incomplete lineage separation from outgroups. Similarly, *Eubacterium tenue*, a species currently assigned to Peptostreptococcaceae, clustered with *Clostridium sordellii* (74% bootstrap support) and was placed in a sister lineage to the Paraclostridium clade, deviating from the core genus-level clades of the family. Notably, *Tepidibacter*, a taxon historically assigned to this family, formed a distinct phylogenetic lineage with 85% bootstrap support, which was clearly separated from the core monophyletic clades of Peptostreptococcaceae in this tree. These core clades were stably composed of the family’s type genus Peptostreptococcus, as well as *Clostridioides*, *Paraclostridium*, and *Peptacetobacter*, with high nodal support (95%–100% bootstrap values). Collectively, these 16S rRNA-based analyses revealed the critical limitations of single-gene phylogeny in resolving fine-scale genus-level relationships within the Peptostreptococcaceae, thereby necessitating genome-wide analyses for accurate phylogenetic inference and taxonomic reclassification.

### Genome-wide phylogenomics of the full protein repertoire: resolving genus-level delineation

**Fig 2:**
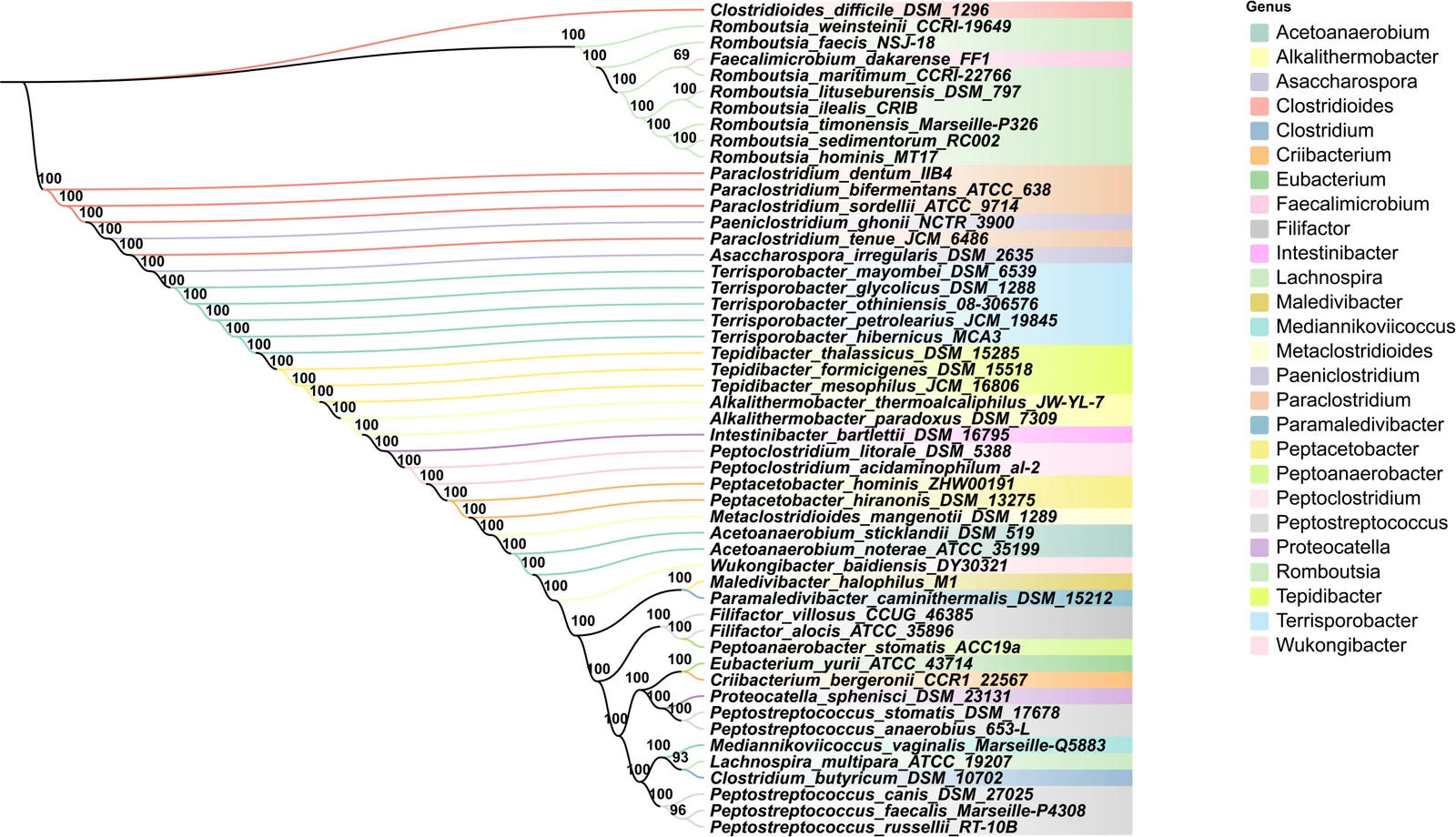
Phylogenetic tree of Peptostreptococcaceae strains based on protein sequences. This figure shows a phylogenetic tree constructed using protein sequences, depicting the phylogenetic relationships between Peptostreptococcaceae strains and their related taxa. The branching pattern reflects evolutionary affinities among different strains, which are inferred from differences in their protein sequences, and the numbers labeled on the branches represent bootstrap support values (based on 1,000 replicates). These values indicate the reliability of each branching node, with values ≥70% typically considered statistically robust.

To overcome the limited resolution of 16S rRNA sequencing for closely related taxa and accurately refine internal phylogenetic relationships within the *Peptostreptococcaceae*, we focused our analysis on a data set of 51 core strains from this family. After identifying reliable single-copy orthologous genes, we concatenated their conserved protein sequences to construct a robust ML phylogenetic tree (Fig. 2). In contrast to the phylum-level 16S rRNA overview (Fig. 1), Fig. 2 resolves genus-level differentiation within the *Peptostreptococcaceae* using multi-locus core protein data. Furthermore, to ensure comparability, a corresponding 16S rRNA phylogenetic tree was reconstructed from the same set of 51 strains (Fig. S1), enabling direct comparison between single-gene and core-protein approaches at equivalent taxonomic depth.

The high-resolution core-protein tree (Fig. 2) reveals three definitive taxonomic patterns. First, current members of the genus *Romboutsia* do not form a monophyletic group. Instead, they disperse across phylogenetically distinct clades with substantial genetic divergence, indicating that the existing taxonomic boundary of *Romboutsia* incorporates evolutionarily heterogeneous species. Second, all classified *Terrisporobacter* species cluster into a tightly unified monophyletic clade with strong bootstrap support (≥90%). This clustering validates the genus as a stable and well-circumscribed taxonomic unit within the *Peptostreptococcaceae*. Third, the genus *Clostridioides*, represented by the model strain *Clostridioides difficile* DSM 1296, forms an independent and highly supported monophyletic branch (bootstrap ≥95%). This branch is clearly separated from the *Romboutsia* and *Terrisporobacter* clades, reinforcing its status as a distinct core genus within the family. Abundant fully supported nodes (bootstrap = 100%) throughout the tree further validate the reliability of the core-protein phylogenetic framework and strengthen the credibility of all genus-level taxonomic inferences. Figure 2 and Fig. S1 collectively demonstrate that genome-wide core protein data sets markedly improve genus-level resolution within the *Peptostreptococcaceae*, yielding well-supported monophyletic delineations that are not recovered by 16S rRNA-based analysis at the same strain scale.

### Conservation analysis of spore proteins: supporting their application as taxonomic markers

**Fig 3:**
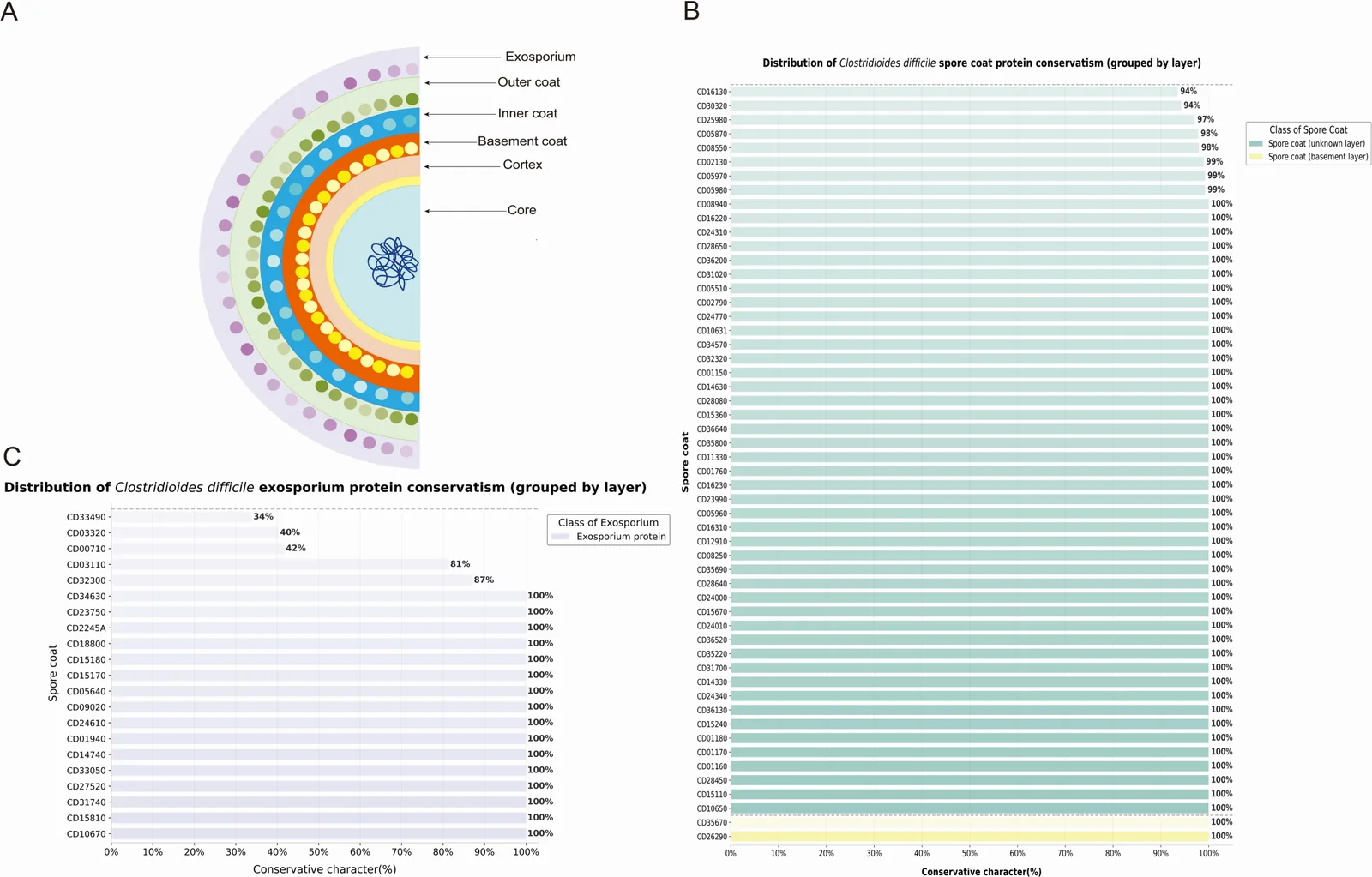
Spore layered structure and conserved distribution of spore-related proteins in *C. difficile.* (**A**) Schematic of the spore hierarchical structure. This panel illustrates the complete layered organization of the spore, including the core (innermost region), cortex (surrounding the core), basement coat, inner coat, outer coat (collectively forming the spore coat), and exosporium (outermost layer). The diagram also indicates the localization of spore-related proteins within these layers. (**B**) Conserved distribution of spore coat proteins (basement layer and unknown layer) in *C. difficile*. The x-axis represents the conservation rate (range: 0–1), calculated as the proportion of tested *C. difficile* strains in which a given spore coat protein was identified as conserved (defined as 100% sequence identity to the reference sequence) relative to the total number of strains. The y-axis lists individual spore coat protein names. Each color-coded bar corresponds to a protein’s conservation rate: yellow (basement layer) and green (unknown layer). (**C**) Conserved distribution of exosporium proteins in *C. difficile*. Analyzed using the same method as panel B, this panel depicts the conservation of proteins localized to the exosporium layer. Each bar represents an individual exosporium protein, with purple indicating the exosporium layer.

Figure 3A illustrates the multilayered structure of the *C. difficile* spore and the distribution of spore coat proteins and exosporium proteins across distinct layers (e.g., basement coat, inner/outer coat, and exosporium)—this structural context justifies analyzing protein conservation based on spatial localization. Using this framework, protein sequences from 280 clinical *C. difficile* isolates were aligned against strain 630’s reference proteome to identify spore coat proteins and exosporium proteins. Following BLASTp filtering of redundant sequences, these proteins exhibited a conservation rate of over 90% across strains (Fig. 3B); notably, 85% of these proteins showed 100% conservation (Fig. 3B and C). Collectively, spore coat proteins displayed significant sequence conservation across *C. difficile* strains, irrespective of their localization within specific layers of the spore structure. Importantly, these conserved spore coat proteins are also present in other sporulating members of the phylum Firmicutes—this cross-generic conservation suggests that they may serve as novel molecular markers for phylogeny within Firmicutes, prompting further investigation of their potential utility.

### Phylogenetics of spore proteins: evaluating genus delimitation

**Fig 4:**
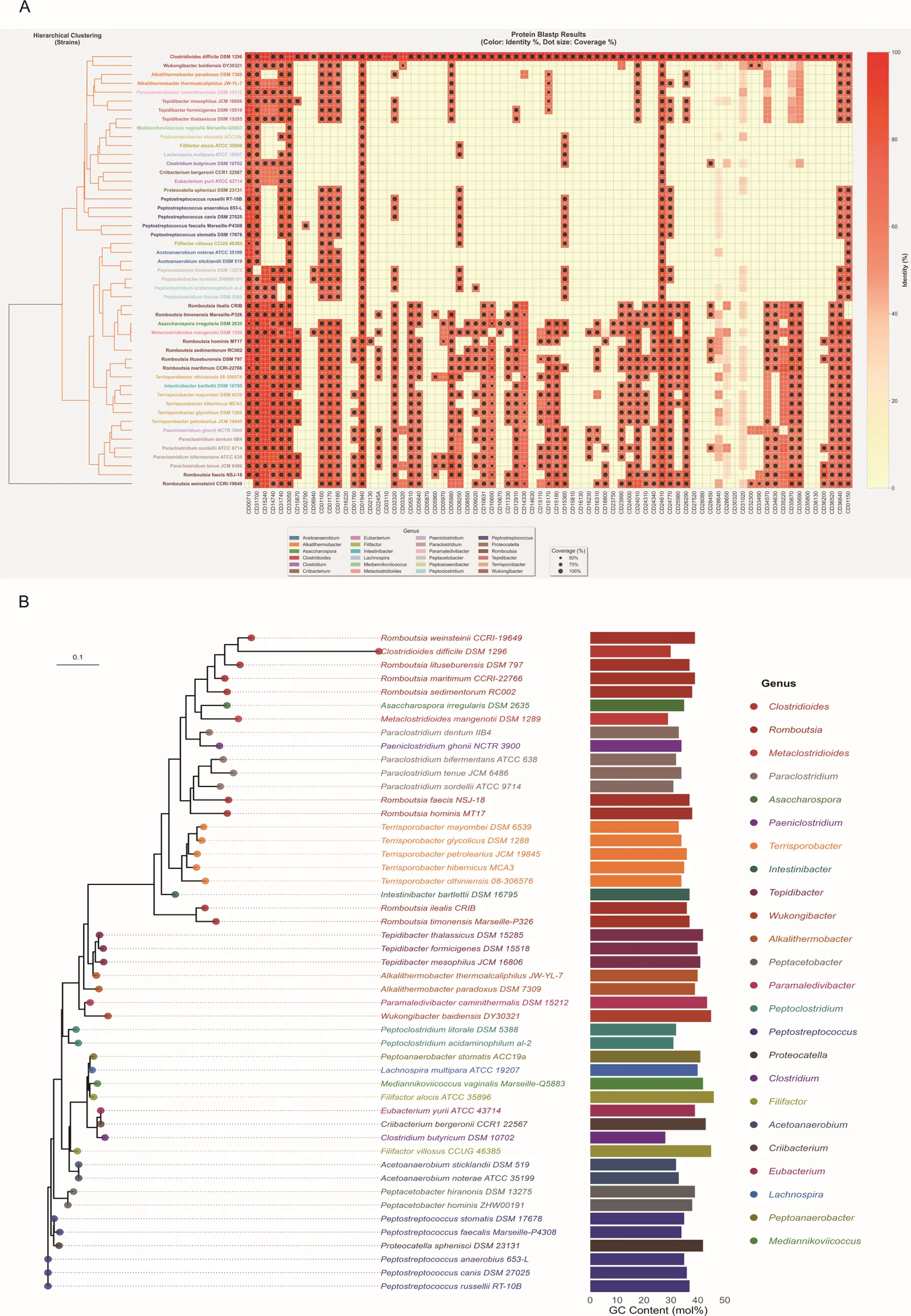
BLASTp alignment results heatmap and neighbor-joining (NJ) phylogenetic tree based on spore/exosporium protein presence/absence matrix. (**A**) Heatmap of BLASTp results. This heatmap presents BLASTp alignment results of strains against target proteins. On the left, a hierarchical clustering dendrogram shows strain clustering derived from sequence identity data, with clustering performed using Euclidean distance and the average linkage method. On the right, a protein similarity heatmap depicts strain-protein sequence similarity, where color intensity represents the identity percentage, and dot size indicates sequence coverage. Strains are grouped by genus and color-coded for genus-level distinction. (**B**) NJ tree based on the presence/absence matrix of 54 spore proteins and 21 exosporium proteins. This phylogenetic tree was constructed via the NJ method based on the presence/absence of 54 spore proteins and 21 exosporium proteins, with different colors corresponding to distinct genera. Supplementary GC content bar charts are included for each strain.

BLASTp alignment results showed high sequence similarity in spore formation-related proteins between *Paraclostridium* and *Metaclostridioides* strains (Fig. 4A). These proteins included key spore formation factors. Among these, SipL functions as a spore assembly protein, and SpoIVA acts as a spore cortex assembly protein. Notably, the gene clusters annotated at the bottom of the heatmap directly correspond to these proteins, a correspondence that ensures accurate identification of the target proteins. Heatmap of BLASTp results (Fig. 4A) revealed clear quantitative characteristics of sequence identity and coverage for the target proteins between the two genera. Specifically, more than 80% of the co-detected spore formation-related proteins exhibited sequence identity ≥80% (corresponding to the orange signal range in the heatmap) and sequence coverage ≥90% (corresponding to the high-coverage markers in the heatmap). This quantitative data confirm the high integrity of the conserved regions of these proteins. Collectively, these quantitative features of the heatmap directly validate high consistency in both sequence and structural integrity of the conserved spore formation-related proteins across the two genera.

Furthermore, the sequence similarity revealed by BLASTp receives strong support from phylogenetic clustering results. Specifically, *Paraclostridium* and *Metaclostridioides* cluster tightly into a single clade in the left hierarchical clustering tree. This clustering result confirms the molecular consistency of the two genera in key spore formation-related conserved domains. It also reinforces their close phylogenetic relationship at the molecular level. In contrast, *Clostridioides* strains (with *Clostridioides difficile* DSM 1296 as the representative) exhibit distinct patterns that match their taxonomic divergence. The sequence similarity in the corresponding region of the heatmap for this genus exceeds 95%. This value is significantly higher than the 80%–100% range observed for the two aforementioned genera. Its sequence coverage also exceeds 95%. Meanwhile, *Clostridioides* forms an independent clade with a distant phylogenetic relationship. These coordinated features collectively indicate substantial differences in spore-related proteins between *Clostridioides* and the two genera mentioned above (*Paraclostridium* and *Metaclostridioides*).

To prioritize the taxonomic value of spore coat proteins and exosporium proteins while avoiding sequence variation-induced biases, we further constructed an NJ phylogenetic tree based on the presence-absence matrix of 54 spore coat proteins and 21 exosporium proteins (Fig. 4B). Phylogenetic analysis of this spore protein-based NJ tree yielded clear taxonomic insights: first, *Paraclostridium* and *Metaclostridioides* belong to the core phylogenetic group of the Peptostreptococcaceae family, sharing a closer phylogenetic relationship with each other than with more distantly related genera and thus forming a tight cluster; second, the genus *Clostridioides* (represented herein by *C. difficile*) exhibits an independent divergence trend, with a topology consistent with the formation of an exclusive monophyletic clade when incorporating additional conspecific strains; third, although *Romboutsia* appears monophyletic in the current tree, marked divergence among its internal subclades and close phylogenetic associations with external genera suggest a potential non-monophyletic status.

### Phylogeny of core spore coat proteins and AAI analysis: delineating genetic boundaries at the genus level

**Fig 5:**
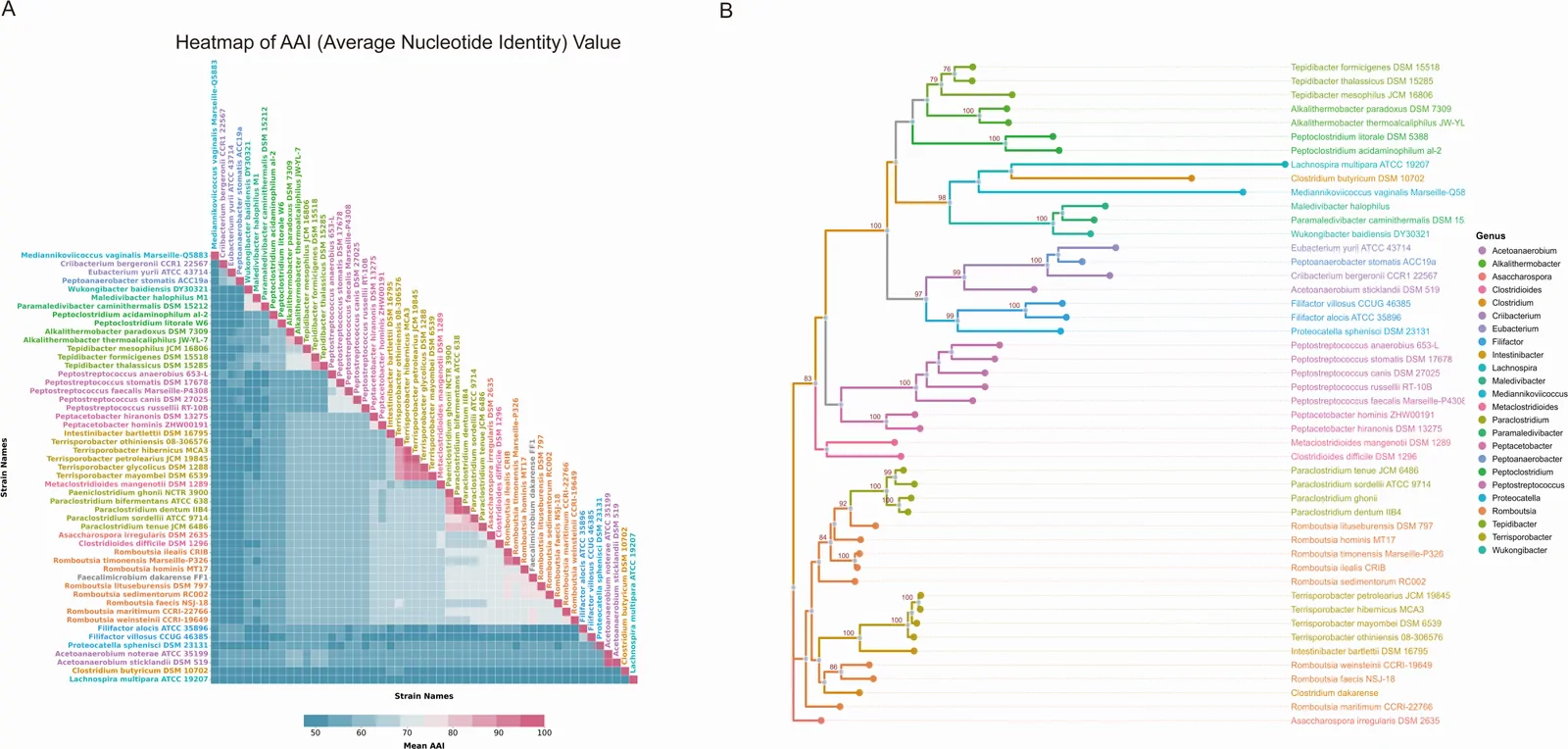
(**A**) Heatmap of average amino acid identity (AAI) results. This heatmap presents AAI values among different microbial strains, which are utilized to analyze the phylogenetic relationships and genomic similarity of the tested strains. (**B**) Maximum likelihood (ML) tree based on the amino acid variation matrix of three core spore coat proteins (SpoIVA, YabG, and CotE). The ML phylogenetic tree was constructed using RAxML, illustrating the evolutionary relationships among different bacterial strains. Branch colors are encoded according to the genus-level classification of strains (see the right-hand legend). Each leaf node represents a bacterial strain, with the full species name displayed as its label. Red numbers on the nodes indicate bootstrap support values (only values > 70 are shown), and branch lengths correspond to evolutionary distances.

To further enhance the robustness of phylogenetic inferences, we first performed AAI analysis to elucidate the genome-wide genetic distance patterns across the tested strains (Fig. 5A). Subsequently, an ML phylogenetic tree was constructed based on three conserved spore coat proteins (SpoIVA, CotE, and YabG; Fig. 5B)—key markers selected for their high conservation and functional relevance in spore assembly across Peptostreptococcaceae. The genome-wide genetic divergence patterns revealed by AAI analysis, together with the topological structure of the ML tree, provide critical evidence for the proposed genus-level taxonomic revisions. Specifically, *Eubacterium tenue* clusters with typical *Paeniclostridium* strains (e.g., *Paeniclostridium ghonii* and *Paeniclostridium sordellii*) to form a high-confidence branch (bootstrap support ≥ 90%), with AAI values within this branch ranging from 70% to 95%—a range that aligns with the widely accepted genetic threshold for genus-level classification (56). To further validate this threshold, we compared it with the intra-genus genetic characteristics of *Terrisporobacter* (mean AAI = 90.68, OF = 75.06): the overlapping AAI ranges between the two groups confirm that the *Eubacterium tenue-Paeniclostridium* branch’s genetic patterns are consistent with genus-level clustering. This key finding directly supports the taxonomic revision of merging *Eubacterium tenue* along with *Paeniclostridium* species into the emended genus *Paraclostridium*, resolving long standing ambiguities in their taxonomic boundaries.

The phylogenetic conclusions based on core spore coat proteins are generally consistent with those from AAI analysis and previous phylogenetic analysis of full spore protein repertoire (includes both core and accessory components), despite minor discrepancies. These results overall support the reliability of taxonomic inferences. The intra-genus AAI values are significantly higher than inter-genus values—for instance, the intra-genus AAI within *Terrisporobacter* is 90.68, which is higher than the AAI values observed between distantly related genera (e.g., 79.17 between *Alkalithermobacter* and other genera, 58.96 in other cross-genus combinations). This AAI pattern corresponds to the topological differentiation of the spore coat protein tree (Fig. 5B), confirming that both molecular data types reflect consistent evolutionary relationships.

In the ML tree (Fig. 5B), *C. difficile* forms a relatively independent monophyletic branch, with a significant genetic distance from adjacent groups (e.g., the genus *Peptoclostridium*)—this topological feature emphasizes the phylogenetic uniqueness of *Clostridioides* as an independent taxonomic unit, strongly supporting the conclusion that it merits independent classification due to its molecular differentiation. Furthermore, within the genus *Romboutsia*, a clear phylogenetic differentiation pattern is observed—consistent with the earlier finding that *Romboutsia* is non-monophyletic: species such as *R. weinstockii* and *R. sedimentorum* cluster tightly, forming the genus’s core group, while *R. ilealis* and *R. timonensis* each form separate independent branches with considerable genetic distance from the core group. Notably, these two species do not form stable clusters with other closely related genera in the Peptostreptococcaceae family (e.g., *Paraclostridium*), supporting their classification as groups with uncertain taxonomic positions within the family.

Two key taxonomic revisions are supported by the ML tree results: first, species of the genus *Paraclostridium* (e.g., *P. ghonii* and *P. sordellii*) form closely clustered, high-support branches in the trees. Notably, *Eubacterium tenue* clusters with this genus’s core group, with bootstrap support values ranging from 92% to 100%; this support is well above the 70% high-confidence threshold in phylogenetic analysis, directly validating the hypothesis of reclassifying *Eubacterium tenue* as *Paraclostridium tenue* and providing reliable phylogenetic evidence for this adjustment. Second, *Alkalithermobacter thermocaliphilus* and *A. paradoxus* form relatively independent monophyletic branches, clustering tightly within their respective branches and exhibiting significant genetic distances from other major clades in the Peptostreptococcaceae family (e.g., *Clostridioides* and *Romboutsia*). No cross-clade clustering is observed, supporting the conclusion that they should be classified into a new genus, *Alkalithermobacterium*, and providing crucial topological evidence for establishing this new genus.

Analyses based on the full spore protein repertoire (Fig. 4B and 5A) reflect overall proteome similarity across strains. In contrast, the ML tree constructed from three core spore proteins (SpoIVA, YabG, and CotE) focuses on highly conserved genes characterized by strict vertical inheritance and slower substitution rates under strong purifying selection. Such conservation makes them well suited for resolving deep phylogenetic relationships at the family level and above but limits their ability to distinguish fine-scale relationships among closely related taxa. For example, *Asaccharospora irregularis* clusters closely with *Metaclostridium mangenotii* and *Paraclostridium tenue* in Fig. 4B and 5A, reflecting the high similarity of their full spore-related protein profiles and genome-wide AAI values (68.2% and 71.5%, respectively). Nevertheless, the core spore coat proteins of *A. irregularis* contain several unique amino acid substitutions and short indels. Despite the overall conservation, such variations are amplified in the three-gene ML tree, resulting in a distinct long branch (Fig. 5B). This topological discrepancy does not contradict the overall phylogenetic signal but is consistent with the 60%–70% AAI threshold for genus delimitation in Peptostreptococcaceae, supporting *A. irregularis* as a well-defined independent genus within the family.

Collectively, core spore protein phylogeny and genome-wide AAI analysis provide generally consistent signals for genus-level delineation, while topological variations highlight the evolutionary distinctiveness of certain lineages. These complementary molecular approaches support the use of spore coat proteins as reliable markers for defining the taxonomic boundaries within the Peptostreptococcaceae family. They also provide a basis for refining its taxonomic framework, including the merger of *Eubacterium* into *Paraclostridium*, the establishment of a new genus for *Alkalithermobacter*, and revisions to the taxonomic status of *Romboutsia* species (10).

### Analysis of spore core proteins and regulatory mechanisms: uncovering the functional basis of conservation

**Fig 6:**
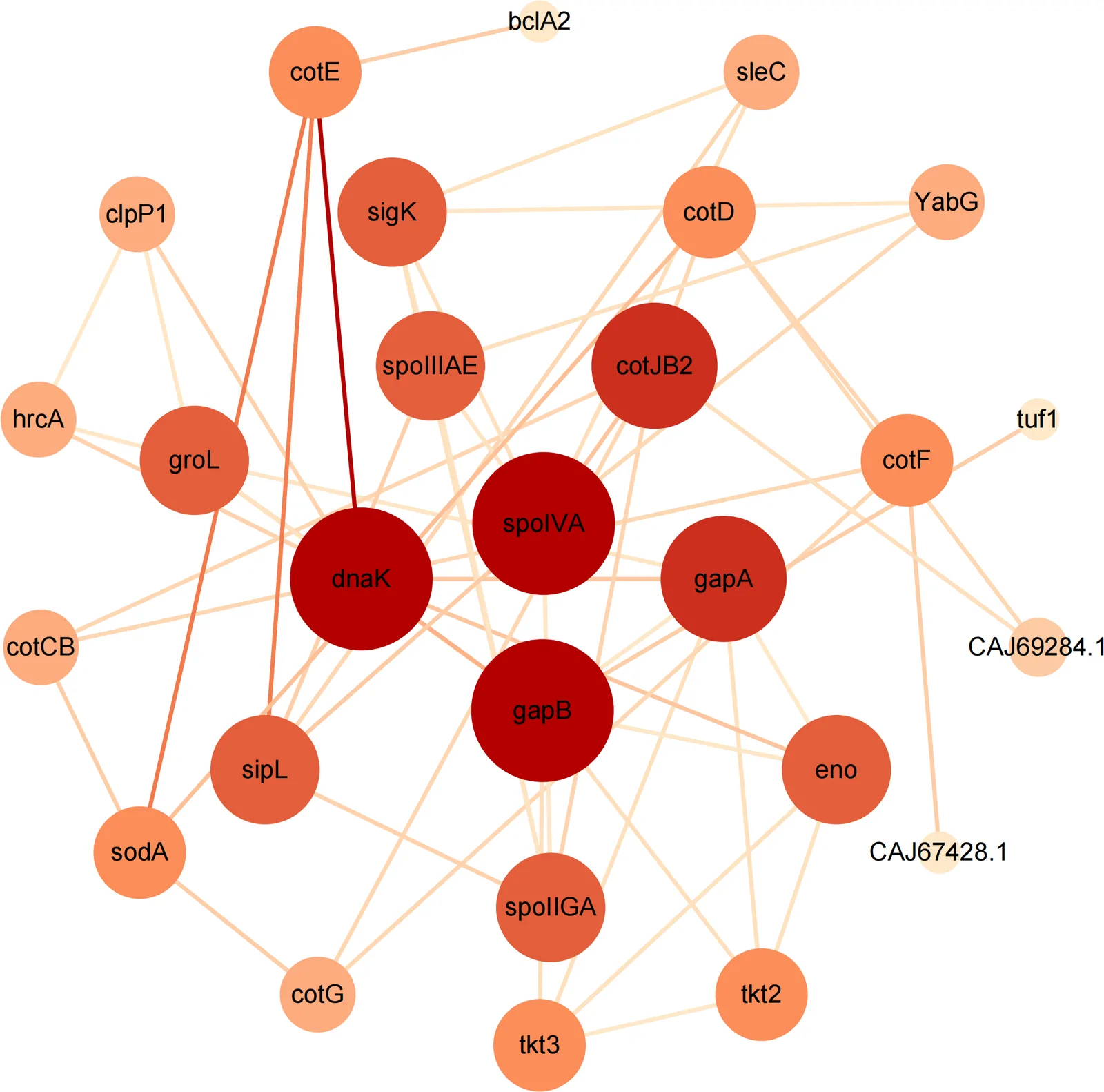
Spore coat/exosporium genes: co-expression PPI network. This gene co-expression PPI network consists of 27 nodes and 98 edges (average degree 6.76). Circular nodes represent spore coat and exosporium genes. Arranged concentrically by connectivity degree, nodes have size and color positively correlating with degree (larger/darker = higher degree); genes with degree > average are defined as core targets. Node color intensity further reflects the co-expression Pearson correlation coefficient (darker = more significant), while edge thickness corresponds to the STRING combined score (0–1; thicker = more reliable interactions).

To explore interaction patterns among the identified spore coat proteins and exosporium proteins, we conducted protein-protein interaction (PPI) analysis using data from the STRING database (Fig. 6). This analysis revealed 58 high-confidence interactions (edges) involving 27 genes (nodes), with an average connectivity degree of 3.87. The STRING combined scores for these interactions ranged from 0.700 to 0.999, and 65.5% of these interactions had a score of ≥0.8, indicating high reliability. Among the genes included in these high-confidence interactions, several core nodes exhibited notable connectivity, including the three conserved spore coat proteins (SpoIVA, CotE, and YabG) highlighted in our phylogenetic analysis. Specifically, *spoIVA* (CD26290) had a connectivity degree of 7, interacting with *YabG* (CD35690), *sipL* (CD35670), and *sleC* (CD05510; interaction scores: 0.728–0.912); *gapA* (CD31740) had a degree of 6; *eno* (CD31700) and *cotE* (CD14330) had degrees of 5 and 4, respectively; and both *clpP1* (CD33050) and *YabG* had a degree of 3. Notably, direct high-confidence interactions were observed between YabG and SpoIVA (score: 0.801) as well as between Eno and GapA (score: 0.987), with the former linking two of the three core spore coat proteins. Collectively, these interactions, particularly those centered on SpoIVA, CotE, and YabG, are likely pivotal for spore coat assembly as they integrate key structural and regulatory components of the assembly machinery.

To further define the functional relevance of these interactions, we classified genes with a connectivity degree exceeding the average (3.87) as core interaction targets, identifying 17 such genes (e.g., *dnaK*, *spoIVA*, *gapA*, *eno*, and *cotE*). Importantly, the three core spore coat proteins (SpoIVA, CotE, and YabG) are among these high-connectivity targets and exhibit the highest conservation within *Peptostreptococcaceae*, consistent with the sequence conservation patterns observed in Fig. 4A. While other core interaction targets display varying levels of conservation across the family, the exceptional conservation of SpoIVA, CotE, and YabG underscores their functional centrality in the spore coat assembly mechanism. This conserved interaction module, which is anchored by the three core proteins, further validates their suitability as robust taxonomic markers for resolving closely related species within *Peptostreptococcaceae*.

## DISCUSSION

Conventional bacterial classification methods rely heavily on phenotypic characteristics, including morphological traits (e.g., cell size, shape, and flagellation) (57), cultural properties (e.g., nutritional requirements and optimal growth temperatures), and biochemical test results. While intuitive, this approach is constrained by subjective interpretation and inherent technical limitations that compromise taxonomic accuracy (58). Modern classification has shifted toward genotypic data from DNA sequence analyses, yet 16S rRNA gene sequencing, still the most widely used single-gene marker, remains limited by gene recombination and mutation that obscure true evolutionary relationships and low sequence divergence among closely related taxa, which restricts resolution and yields ambiguous assignments (59). To address these longstanding limitations, this study adopted a polyphasic taxonomic framework integrating the strengths of genome-scale precision and functional marker reliability. Specifically, we synthesized whole-genome single-copy orthologous protein sequence data, supplemented with AAI calculations, and further validated these findings through cross-verification using spore proteins as taxonomic markers. This integrated strategy directly overcomes the shortcomings of traditional phenotypic approaches and single-locus genotypic methods, with whole-genome single-copy orthologous protein analysis as the core foundational component.

Phylogenetic analysis of whole-genome single-copy orthologous proteins has become a cornerstone of modern microbial taxonomy, with enhanced resolution for bacterial classification (60). These proteins are uniquely suited for phylogenetic inference due to their functional constraints, as they rarely undergo recombination, accumulate mutations at stable, clock-like rates, and retain sufficient divergence to distinguish closely related groups. Consistent with these inherent advantages, our whole-genome protein phylogeny (Fig. 2) clearly resolved core clustering patterns of key genera within Peptostreptococcaceae without conflicting signals. For instance, it confirmed the tight phylogenetic affinity between *Paraclostridium* and *Metaclostridioides*, the distinct evolutionary position of *Clostridioides*, and the putative non-monophyletic status of *Romboutsia*. These results align with recent taxonomic revisions based on comparative genomics (10), further validating the reliability of our foundational framework.

To further strengthen this framework, we integrated AAI calculations, a robust metric for quantifying genomic similarity that independently validates taxonomic boundaries (61). AAI is widely adopted for genus-level classification, with a threshold of ≥65%–70% typically defining members of the same genus (62). Our AAI data strongly corroborated phylogenetic insights from the single-copy protein tree, as the mean AAI of 58.96 between *Alkalithermobacter* and *Peptacetobacter* fell well below this threshold, reinforcing their distinct generic status. Additionally, genetic distance data from the whole-genome protein tree showed the distance between the *Clostridioides difficile* DSM 1296 clade and the *Metaclostridioides* clade was approximately 0.9, far exceeding the typical intra-genus divergence threshold (≤0.05 for most bacterial genera). This convergence of phylogenetic topology, genetic distance, and AAI evidence, supported by extensive prior literature, underscores the rigor and robustness of our whole-genome-based foundational framework.

Building on this reliable whole-genome framework, we further integrated conserved spore coat/exosporium protein sequences into the polyphasic taxonomic strategy. Notably, our findings regarding *Terrisporobacter* are highly consistent with prior studies indicating this genus belongs to the Peptostreptococcaceae *sensu stricto* monophyletic clade (10). This consistency confirms *Terrisporobacter*’s core taxonomic position within this family and reinforces the reliability of our integrated framework.

Employing spore coat proteins as specialized molecular markers yielded critical taxonomic insights, complementing genome-scale analyses. For instance, it accurately assigned *C. difficile* to the genus *Clostridioides* and phylogenetically distinguished the non-pathogenic *Metaclostridioides mangenotii* from this taxon. The molecular divergence observed in spore coat proteins provides direct evidence for *Clostridioides*’ taxonomic distinctiveness from its close relatives, supporting its status as a phylogenetically robust independent unit. Intergeneric genetic differences revealed by AAI and whole-genome analyses further corroborate clear genetic boundaries within Peptostreptococcaceae, aligning with inferences from spore protein data. Together, these lines of evidence, including spore coat protein divergence and congruent genome-scale genetic signals, validate the phylogenetic independence of *Clostridioides*, consistent with prior observations that *C. difficile* harbors spore-associated sequence specificity driven by unique genetic events.

The conserved nature of spore coat proteins, a key factor in their taxonomic utility, stems from functional constraints imposed by their role in spore assembly and survival. Specifically, spore coat assembly relies on specific PPIs, so a single protein cannot evolve independently without disrupting its obligate partners, thereby reducing mutation likelihood. This constraint contrasts sharply with *C. difficile* toxins such as TcdA and TcdB, which evolve in response to environmental factors like host microenvironments and exhibit high variability via recombination (63), highlighting the superiority of spore coat proteins as stable taxonomic markers. Furthermore, the spore coat’s function as a barrier against enzymatic degradation and antibiotics (64) is thought to be linked to its assembly, which may be controlled by a protein homeostasis network previously shown to maintain protein integrity in related systems.

This conserved assembly process is regulated by a protein homeostasis network centered on the *dnaK-groL* chaperone system and the *clpP1* protease. For instance, *clpP1* interacts with both *dnaK* and *groL*, with interaction scores ranging from 0.775 to 0.875. This regulatory network ensures proper folding and quality control of spore coat proteins, as supported by previous studies (65–68). Phylogenetic analyses based on spore coat proteins, together with 16S rRNA sequencing, AAI calculations, and ML/NJ tree constructions, established consistent taxonomic signals validating the phylogenetic utility of spore coat proteins. Meanwhile, high-confidence PPI patterns, conserved core targets, and the underlying regulatory network provided a mechanistic basis for their sequence conservation, a key prerequisite for reliable taxonomic markers. Together, these complementary lines of evidence, namely systematic phylogenetic congruence and robust functional and network conservation, strongly support establishing spore coat proteins as a novel molecular marker for Peptostreptococcaceae taxonomy.

Notably, taxonomic inferences from spore protein-based phylogenies are highly congruent with those derived from the whole-genome protein phylogeny (Fig. 2), with no conflicting signals in the core clustering patterns of key genera. This cross-validation between two distinct marker systems, spore-specific proteins and whole-genome proteins, directly corroborates the reliability of spore protein-based classification inferences. Furthermore, the spore protein-based NJ tree exhibited robust bootstrap support (≥70%) for core branches, and genetic distance data from whole-genome analyses further reinforced the tree’s ability to resolve closely related genera. Collectively, the high congruence between spore protein and whole-genome protein phylogenies, together with corroborative genetic distance evidence from Fig. 2, unequivocally demonstrates the feasibility of spore-associated proteins as credible taxonomic markers for Peptostreptococcaceae and related spore-forming bacteria.

Our study also directly addresses unresolved questions in Peptostreptococcaceae taxonomy and spore biology. Specifically, our comparative analysis identified 11 highly conserved proteins across 51 bacterial species, an area hindered by unknown proteins and unclear layer composition (69). This set may include uncharacterized morphogenetic proteins and provides valuable targets for future spore assembly studies. To enhance genus- and species-level classification, we propose exploring additional core conserved proteins such as single-copy housekeeping proteins with stable evolutionary rates. This expansion will address limitations of single markers, such as 16S rRNA genes, and support efforts to revise *Romboutsia*’s generic boundaries and verify *Terrisporobacter*’s taxonomic status.

In summary, our polyphasic taxonomic framework, anchored in the rigor of whole-genome single-copy orthologous protein phylogeny and AAI calculations and augmented by the novel application of spore coat proteins, overcomes limitations of traditional nucleic acid methods by leveraging a three-dimensional framework encompassing structural conservatism, functional relevance, and ecological adaptability, with each dimension supported by our integrated data. Practically, this approach has resolved genus-level controversies surrounding *C. difficile* and confirmed *M. mangenotii*’s independent evolutionary status, while paving new avenues for taxonomic studies of spore-forming genera such as *Bacillus* and *Clostridioides* and refining microbial classification systems within the phylum Firmicutes. Furthermore, our results are consistent with prior conclusions from concatenated core protein trees, validating that functionally conserved spore coat proteins ensure phylogenetic stability and reinforce taxonomic placements within Peptostreptococcaceae.

## SUPPLEMENTAL MATERIAL

Fig. S1 spectrum.04185-25-s0001.tiff 16S rRNA phylogenetic tree constructed using the same 51 Peptostreptococcaceae strains as those employed for the phylogenomic tree based on concatenated sequences of genome-wide single-copy orthologous proteins in Figure 2.

Fig. S2 spectrum.04185-25-s0002.tiff Full bootstrap support values for all nodes of the maximum likelihood phylogenetic tree corresponding to main Figure 5B.

Supplemental material spectrum.04185-25-s0003.docx Supplemental figure captions.

Table S1 spectrum.04185-25-s0004.xlsx Genomes used in this work, retrieved from NCBIs FTP server.

Table S2 spectrum.04185-25-s0005.xlsx 16S rRNA sequence data used in this work, retrieved from NCBIs FTP server.

Table S3 spectrum.04185-25-s0006.xlsx Genomes used in this work, retrieved from NCBIs FTP server.

Table S4 spectrum.04185-25-s0007.xlsx List of single-copy orthologous proteins used for whole-genome phylogenetic tree construction.

ASM does not own the copyrights to Supplemental Material that may be linked to, or accessed through, an article. The authors have granted ASM a non-exclusive, world-wide license to publish the Supplemental Material files. Please contact the corresponding author directly for reuse.

## References

[1] Won S, Cho S, Kim H. 2024. rRNA operon improves species-level classification of bacteria and microbial community analysis compared to 16S rRNA. Microbiol Spectr 12:e0093124. doi:10.1128/spectrum.00931-24 39365049 PMC11537084

[2] Aggarwal N, Kitano S, Puah GRY, Kittelmann S, Hwang IY, Chang MW. 2023. Microbiome and human health: current understanding, engineering, and enabling technologies. Chem Rev 123:31–72. doi:10.1021/acs.chemrev.2c00431 36317983 PMC9837825

[3] Dommann J, Kerbl-Knapp J, Albertos Torres D, Egli A, Keiser J, Schneeberger PHH. 2024. A novel barcoded nanopore sequencing workflow of high-quality, full-length bacterial 16S amplicons for taxonomic annotation of bacterial isolates and complex microbial communities. mSystems 9:e0085924. doi:10.1128/msystems.00859-24 39254034 PMC11494973

[4] Petrella LA, Sambol SP, Cheknis A, Nagaro K, Kean Y, Sears PS, Babakhani F, Johnson S, Gerding DN. 2012. Decreased cure and increased recurrence rates for *Clostridium difficile* infection caused by the epidemic *C. difficile* BI strain. Clin Infect Dis 55:351–357. doi:10.1093/cid/cis430 22523271 PMC3491778

[5] Desai K, Gupta SB, Dubberke ER, Prabhu VS, Browne C, Mast TC. 2016. Epidemiological and economic burden of *Clostridium difficile* in the United States: estimates from a modeling approach. BMC Infect Dis 16:303. doi:10.1186/s12879-016-1610-3 27316794 PMC4912810

[6] Guh AY, Mu Y, Winston LG, Johnston H, Olson D, Farley MM, Wilson LE, Holzbauer SM, Phipps EC, Dumyati GK, Beldavs ZG, Kainer MA, Karlsson M, Gerding DN, McDonald LC, Emerging Infections Program *Clostridioides difficile* Infection Working Group. 2020. Trends in U.S. burden of *Clostridioides difficile* infection and outcomes. N Engl J Med 382:1320–1330. doi:10.1056/NEJMoa1910215 32242357 PMC7861882

[7] Bi X, Zheng L, Yang Z, Lv T, Tong X, Chen Y. 2023. Retrospective study of the epidemiology of *Clostridioides difficile* infection in the neurosurgery department of a tertiary hospital in China. Infect Drug Resist 16:545–554. doi:10.2147/IDR.S397544 36726387 PMC9885874

[8] Cui Y, Dong D, Zhang L, Wang D, Jiang C, Ni Q, Wang C, Mao E, Peng Y. 2019. Risk factors for *Clostridioides difficile* infection and colonization among patients admitted to an intensive care unit in Shanghai, China. BMC Infect Dis 19:961. doi:10.1186/s12879-019-4603-1 31711425 PMC6849324

[9] Lawson PA, Citron DM, Tyrrell KL, Finegold SM. 2016. Reclassification of *Clostridium difficile* as *Clostridioides difficile* (Hall and O’Toole 1935) Prévot 1938. Anaerobe 40:95–99. doi:10.1016/j.anaerobe.2016.06.008 27370902

[10] Bello S, McQuay S, Rudra B, Gupta RS. 2024. Robust demarcation of the family Peptostreptococcaceae and its main genera based on phylogenomic studies and taxon-specific molecular markers. Int J Syst Evol Microbiol 74. doi:10.1099/ijsem.0.006247 38319314

[11] Cruz-Morales P, Orellana CA, Moutafis G, Moonen G, Rincon G, Nielsen LK, Marcellin E. 2019. Revisiting the evolution and taxonomy of Clostridia, a phylogenomic update. Genome Biol Evol 11:2035–2044. doi:10.1093/gbe/evz096 31076745 PMC6656338

[12] Galperin MY, Brover V, Tolstoy I, Yutin N. 2016. Phylogenomic analysis of the family Peptostreptococcaceae (*Clostridium* cluster XI) and proposal for reclassification of *Clostridium litorale* (Fendrich et al. 1991) and *Eubacterium acidaminophilum* (Zindel et al. 1989) as *Peptoclostridium litorale* gen. nov. comb. nov. and *Peptoclostridium acidaminophilum* comb. nov. Int J Syst Evol Microbiol 66:5506–5513. doi:10.1099/ijsem.0.001548 27902180 PMC5244501

[13] García-Sánchez JE, García-Sánchez E, García-Moro M. 2016. The clinical microbiologist before the taxonomic changes in the genus *Clostridium*. Rev Esp Quimioter 29:239–243. 27628950

[14] Cantos-Parra E, Ramió-Pujol S, Colprim J, Puig S, Bañeras L. 2018. Specific detection of “*Clostridium autoethanogenum*”, *Clostridium ljungdahlii* and *Clostridium carboxidivorans* in complex bioreactor samples. FEMS Microbiol Lett 365. doi:10.1093/femsle/fny191 30084932

[15] Porwal S, Lal S, Cheema S, Kalia VC. 2009. Phylogeny in aid of the present and novel microbial lineages: diversity in *Bacillus*. PLoS One 4:e4438. doi:10.1371/journal.pone.0004438 19212464 PMC2639701

[16] Scaria J, Suzuki H, Ptak CP, Chen JW, Zhu Y, Guo XK, Chang YF. 2015. Comparative genomic and phenomic analysis of *Clostridium difficile* and *Clostridium sordellii*, two related pathogens with differing host tissue preference. BMC Genomics 16:448. doi:10.1186/s12864-015-1663-5 26059449 PMC4462011

[17] Kamboj M, McMillen T, Syed M, Chow HY, Jani K, Aslam A, Brite J, Fanelli B, Hasan NA, Dadlani M, Westblade L, Zehir A, Simon M, Babady NE. 2021. Evaluation of a combined multilocus sequence typing and whole-genome sequencing two-step algorithm for routine typing of *Clostridioides difficile*. J Clin Microbiol 59:e01955-20. doi:10.1128/JCM.01955-20 33177119 PMC8111118

[18] Galperin MY, Yutin N, Wolf YI, Vera Alvarez R, Koonin EV. 2022. Conservation and evolution of the sporulation gene set in diverse members of the *Firmicutes* J Bacteriol 204:e0007922. doi:10.1128/jb.00079-22 35638784 PMC9210971

[19] O’Leary NA, Wright MW, Brister JR, Ciufo S, Haddad D, McVeigh R, Rajput B, Robbertse B, Smith-White B, Ako-Adjei D, et al.. 2016. Reference sequence (RefSeq) database at NCBI: current status, taxonomic expansion, and functional annotation. Nucleic Acids Res 44:D733–45. doi:10.1093/nar/gkv1189 26553804 PMC4702849

[20] Leinonen R, Sugawara H, Shumway M, International Nucleotide Sequence Database C. 2011. The sequence read archive. Nucleic Acids Res 39:D19–D21. doi:10.1093/nar/gkq1019 21062823 PMC3013647

[21] Andrews S. 2010. FastQC: a quality control tool for high throughput sequence data. Babraham Bioinformatics. https://www.bioinformatics.babraham.ac.uk/projects/fastqc.

[22] Bolger AM, Lohse M, Usadel B. 2014. Trimmomatic: a flexible trimmer for Illumina sequence data. Bioinformatics 30:2114–2120. doi:10.1093/bioinformatics/btu170 24695404 PMC4103590

[23] Bankevich A, Nurk S, Antipov D, Gurevich AA, Dvorkin M, Kulikov AS, Lesin VM, Nikolenko SI, Pham S, Prjibelski AD, Pyshkin AV, Sirotkin AV, Vyahhi N, Tesler G, Alekseyev MA, Pevzner PA. 2012. SPAdes: a new genome assembly algorithm and its applications to single-cell sequencing. J Comput Biol 19:455–477. doi:10.1089/cmb.2012.0021 22506599 PMC3342519

[24] Fu L, Niu B, Zhu Z, Wu S, Li W. 2012. CD-HIT: accelerated for clustering the next-generation sequencing data. Bioinformatics 28:3150–3152. doi:10.1093/bioinformatics/bts565 23060610 PMC3516142

[25] Seemann T. 2014. Prokka: rapid prokaryotic genome annotation. Bioinformatics 30:2068–2069. doi:10.1093/bioinformatics/btu153 24642063

[26] Sayers EW, Cavanaugh M, Frisse L, Pruitt KD, Schneider VA, Underwood BA, Yankie L, Karsch-Mizrachi I. 2025. GenBank 2025 update. Nucleic Acids Res 53:D56–D61. doi:10.1093/nar/gkae1114 39558184 PMC11701615

[27] Katoh K, Standley DM. 2013. MAFFT multiple sequence alignment software version 7: improvements in performance and usability. Mol Biol Evol 30:772–780. doi:10.1093/molbev/mst010 23329690 PMC3603318

[28] Kalyaanamoorthy S, Minh BQ, Wong TKF, von Haeseler A, Jermiin LS. 2017. ModelFinder: fast model selection for accurate phylogenetic estimates. Nat Methods 14:587–589. doi:10.1038/nmeth.4285 28481363 PMC5453245

[29] Minh BQ, Schmidt HA, Chernomor O, Schrempf D, Woodhams MD, von Haeseler A, Lanfear R. 2020. IQ-TREE 2: new models and efficient methods for phylogenetic inference in the genomic era. Mol Biol Evol 37:1530–1534. doi:10.1093/molbev/msaa015 32011700 PMC7182206

[30] Emms DM, Kelly S. 2019. OrthoFinder: phylogenetic orthology inference for comparative genomics. Genome Biol 20:238. doi:10.1186/s13059-019-1832-y 31727128 PMC6857279

[31] Buchfink B, Reuter K, Drost HG. 2021. Sensitive protein alignments at tree-of-life scale using DIAMOND. Nat Methods 18:366–368. doi:10.1038/s41592-021-01101-x 33828273 PMC8026399

[32] Buchfink B, Xie C, Huson DH. 2015. Fast and sensitive protein alignment using DIAMOND. Nat Methods 12:59–60. doi:10.1038/nmeth.3176 25402007

[33] Edgar RC. 2004. MUSCLE: multiple sequence alignment with high accuracy and high throughput. Nucleic Acids Res 32:1792–1797. doi:10.1093/nar/gkh340 15034147 PMC390337

[34] Talavera G, Castresana J. 2007. Improvement of phylogenies after removing divergent and ambiguously aligned blocks from protein sequence alignments. Syst Biol 56:564–577. doi:10.1080/10635150701472164 17654362

[35] Shen W, Le S, Li Y, Hu F. 2016. SeqKit: a cross-platform and ultrafast toolkit for FASTA/Q file manipulation. PLoS One 11:e0163962. doi:10.1371/journal.pone.0163962 27706213 PMC5051824

[36] Xie J, Chen Y, Cai G, Cai R, Hu Z, Wang H. 2023. Tree visualization by one table (tvBOT): a web application for visualizing, modifying and annotating phylogenetic trees. Nucleic Acids Res 51:W587–W592. doi:10.1093/nar/gkad359 37144476 PMC10320113

[37] Kumar S, Stecher G, Li M, Knyaz C, Tamura K. 2018. MEGA X: molecular evolutionary genetics analysis across computing platforms. Mol Biol Evol 35:1547–1549. doi:10.1093/molbev/msy096 29722887 PMC5967553

[38] Team RC. 2024. R: a language and environment for statistical computing, r foundation for statistical computing. https://www.R-project.org.

[39] Wickham H. 2016. Ggplot2: elegant graphics for data analysis. Springer-Verlag, Berlin.

[40] Wickham H. 2014. Tidy data. J Stat Soft 59:1–23. doi:10.18637/jss.v059.i10

[41] Paradis E, Schliep K. 2019. Ape 5.0: an environment for modern phylogenetics and evolutionary analyses in R. Bioinformatics 35:526–528. doi:10.1093/bioinformatics/bty633 30016406

[42] Wang L-G, Lam TT-Y, Xu S, Dai Z, Zhou L, Feng T, Guo P, Dunn CW, Jones BR, Bradley T, Zhu H, Guan Y, Jiang Y, Yu G. 2020. Treeio: An R package for phylogenetic tree input and output with richly annotated and associated data. Mol Biol Evol 37:599–603. doi:10.1093/molbev/msz240 31633786 PMC6993851

[43] Capella-Gutiérrez S, Silla-Martínez JM, Gabaldón T. 2009. trimAl: a tool for automated alignment trimming in large-scale phylogenetic analyses. Bioinformatics 25:1972–1973. doi:10.1093/bioinformatics/btp348 19505945 PMC2712344

[44] Clamp M, Cuff J, Searle SM, Barton GJ. 2004. The Jalview Java alignment editor. Bioinformatics 20:426–427. doi:10.1093/bioinformatics/btg430 14960472

[45] Kozlov AM, Darriba D, Flouri T, Morel B, Stamatakis A. 2019. RAxML-NG: a fast, scalable and user-friendly tool for maximum likelihood phylogenetic inference. Bioinformatics 35:4453–4455. doi:10.1093/bioinformatics/btz305 31070718 PMC6821337

[46] Le SQ, Gascuel O. 2008. An improved general amino acid replacement matrix. Mol Biol Evol 25:1307–1320. doi:10.1093/molbev/msn067 18367465

[47] Yang Z. 1994. Maximum likelihood phylogenetic estimation from DNA sequences with variable rates over sites: approximate methods. J Mol Evol 39:306–314. doi:10.1007/BF00160154 7932792

[48] Parks DH, Imelfort M, Skennerton CT, Hugenholtz P, Tyson GW. 2015. CheckM: assessing the quality of microbial genomes recovered from isolates, single cells, and metagenomes. Genome Res 25:1043–1055. doi:10.1101/gr.186072.114 25977477 PMC4484387

[49] Hyatt D, Chen GL, Locascio PF, Land ML, Larimer FW, Hauser LJ. 2010. Prodigal: prokaryotic gene recognition and translation initiation site identification. BMC Bioinformatics 11:119. doi:10.1186/1471-2105-11-119 20211023 PMC2848648

[50] Konstantinidis KT, Tiedje JM. 2005. Towards a genome-based taxonomy for prokaryotes. J Bacteriol 187:6258–6264. doi:10.1128/JB.187.18.6258-6264.2005 16159757 PMC1236649

[51] Konstantinidis KT, Rosselló-Móra R, Amann R. 2017. Uncultivated microbes in need of their own taxonomy. ISME J 11:2399–2406. doi:10.1038/ismej.2017.113 28731467 PMC5649169

[52] Szklarczyk D, Kirsch R, Koutrouli M, Nastou K, Mehryary F, Hachilif R, Gable AL, Fang T, Doncheva NT, Pyysalo S, Bork P, Jensen LJ, von Mering C. 2023. The STRING database in 2023: protein-protein association networks and functional enrichment analyses for any sequenced genome of interest. Nucleic Acids Res 51:D638–D646. doi:10.1093/nar/gkac1000 36370105 PMC9825434

[53] Shannon P, Markiel A, Ozier O, Baliga NS, Wang JT, Ramage D, Amin N, Schwikowski B, Ideker T. 2003. Cytoscape: a software environment for integrated models of biomolecular interaction networks. Genome Res 13:2498–2504. doi:10.1101/gr.1239303 14597658 PMC403769

[54] Parte AC, Sardà Carbasse J, Meier-Kolthoff JP, Reimer LC, Göker M. 2020. List of prokaryotic names with standing in nomenclature (LPSN) moves to the DSMZ. Int J Syst Evol Microbiol 70:5607–5612. doi:10.1099/ijsem.0.004332 32701423 PMC7723251

[55] Parks DH, Chuvochina M, Waite DW, Rinke C, Skarshewski A, Chaumeil PA, Hugenholtz P. 2018. A standardized bacterial taxonomy based on genome phylogeny substantially revises the tree of life. Nat Biotechnol 36:996–1004. doi:10.1038/nbt.4229 30148503

[56] Lee PG, Yin L, Wei X, Shi J, Masuyer G, Wentz TG, Chen P, Xu Y, Liang J, Zhang H, Persson Košenina S, Lobb B, Mansfield M, Gill SS, Pellett S, Stenmark P, Doxey AC, Dong M. 2025. Identification and characterization of botulinum neurotoxin-like two-component toxins in *Paeniclostridium ghonii*. Sci Adv 11:eadx6145. doi:10.1126/sciadv.adx6145 41223264 PMC12609057

[57] Jiao J-Y, Abdugheni R, Zhang D-F, Ahmed I, Ali M, Chuvochina M, Dedysh SN, Dong X, Göker M, Hedlund BP, et al.. 2024. Advancements in prokaryotic systematics and the role of Bergey’s International Society for Microbial Systematicsin addressing challenges in the meta-data era. Natl Sci Rev 11. doi:10.1093/nsr/nwae168 PMC11275469 39071100

[58] Freitas DB, Reis MP, Lima-Bittencourt CI, Costa PS, Assis PS, Chartone-Souza E, Nascimento AMA. 2008. Genotypic and phenotypic diversity of *Bacillus* spp. isolated from steel plant waste. BMC Res Notes 1:92. doi:10.1186/1756-0500-1-92 18928552 PMC2588453

[59] Liu Y, Lai Q, Göker M, Meier-Kolthoff JP, Wang M, Sun Y, Wang L, Shao Z. 2015. Genomic insights into the taxonomic status of the *Bacillus cereus* group. Sci Rep 5:14082. doi:10.1038/srep14082 26373441 PMC4571650

[60] Reiter L, Tourasse NJ, Fouet A, Loll R, Davison S, Økstad OA, Piehler AP, Kolstø A-B. 2011. Evolutionary history and functional characterization of three large genes involved in sporulation in *Bacillus cereus* group bacteria. J Bacteriol 193:5420–5430. doi:10.1128/JB.05309-11 21821775 PMC3187416

[61] Auch AF, von Jan M, Klenk H-P, Göker M. 2010. Digital DNA-DNA hybridization for microbial species delineation by means of genome-to-genome sequence comparison. Stand Genomic Sci 2:117–134. doi:10.4056/sigs.531120 21304684 PMC3035253

[62] Zimenkov D, Ushtanit A. 2025. Comparative analysis of evolutionary distances using the genus *Mycobacterium* Int J Mol Sci 26:10471. doi:10.3390/ijms262110471 41226510 PMC12607691

[63] Mansfield MJ, Tremblay BJ-M, Zeng J, Wei X, Hodgins H, Worley J, Bry L, Dong M, Doxey AC. 2020. Phylogenomics of 8,839 *Clostridioides difficile* genomes reveals recombination-driven evolution and diversification of toxin A and B. PLoS Pathog 16:e1009181. doi:10.1371/journal.ppat.1009181 33370413 PMC7853461

[64] Zeng J, Wang H, Dong M, Tian GB. 2022. Spore: coat assembly and formation. Emerg Microbes Infect 11:2340–2349. doi:10.1080/22221751.2022.2119168 36032037 PMC9542656

[65] Seydlová G, Halada P, Fišer R, Toman O, Ulrych A, Svobodová J. 2012. DnaK and GroEL chaperones are recruited to the *Bacillus subtilis* membrane after short-term ethanol stress. J Appl Microbiol 112:765–774. doi:10.1111/j.1365-2672.2012.05238.x 22268681

[66] Secaira-Morocho H, Castillo JA, Driks A. 2020. Diversity and evolutionary dynamics of spore-coat proteins in spore-forming species of Bacillales. Microb Genom 6:mgen000451. doi:10.1099/mgen.0.000451 33052805 PMC7725329

[67] Calloni G, Chen T, Schermann SM, Chang HC, Genevaux P, Agostini F, Tartaglia GG, Hayer-Hartl M, Hartl FU. 2012. DnaK functions as a central hub in the *E. coli* chaperone network. Cell Rep 1:251–264. doi:10.1016/j.celrep.2011.12.007 22832197

[68] Liu S, Lei T, Tan Y, Huang X, Zhao W, Zou H, Su J, Zeng J, Zeng H. 2025. Discovery, structural characteristics and evolutionary analyses of functional domains in *Acinetobacter baumannii* phage tail fiber/spike proteins. BMC Microbiol 25:73. doi:10.1186/s12866-025-03790-2 39939914 PMC11823257

[69] Bauda E, Gallet B, Moravcova J, Effantin G, Chan H, Novacek J, Jouneau PH, Rodrigues CDA, Schoehn G, Moriscot C, Morlot C. 2024. Ultrastructure of macromolecular assemblies contributing to bacterial spore resistance revealed by *in situ* cryo-electron tomography. Nat Commun 15:1376. doi:10.1038/s41467-024-45770-6 38355696 PMC10867305

