# Supplementary figures and images for: Reliable delineation of Clostridioides difficile and related members of the family Peptostreptococcaceae using phylogenomics and spore coat protein-specific molecular markers
Source: Microbiol Spectr. 2026 Jun 15;14(7):e04185-25. doi: 10.1128/spectrum.04185-25 (PMC13339974; doi:10.1128/spectrum.04185-25)

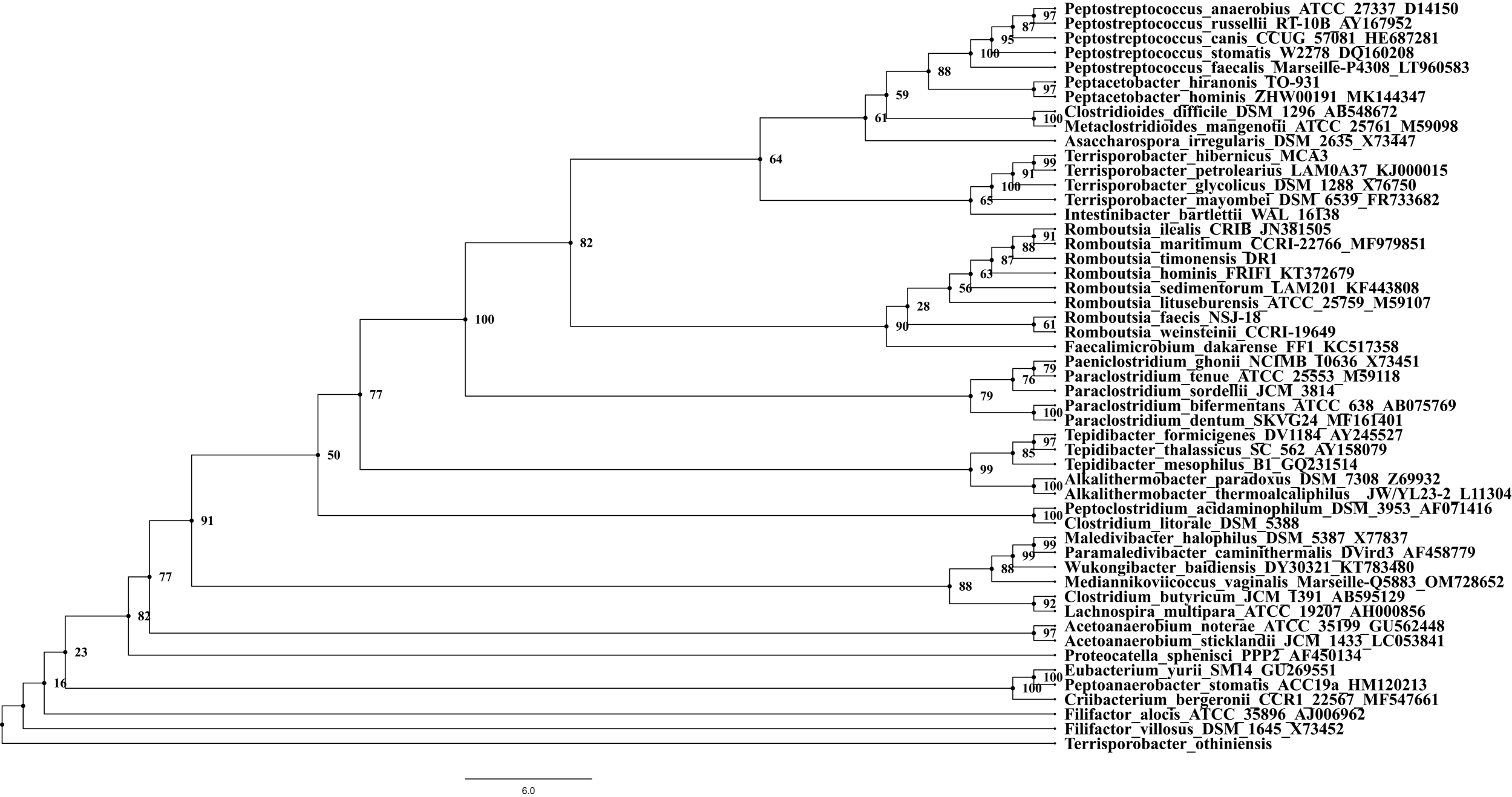

Supplement: Fig. S1 — 16S rRNA phylogenetic tree constructed using the same 51 Peptostreptococcaceae strains as those employed for the phylogenomic tree based on concatenated sequences of genome-wide single-copy orthologous proteins in Figure 2. [file spectrum.04185-25-s0001.tiff]
