# Supplementary material for: Reliable delineation of Clostridioides difficile and related members of the family Peptostreptococcaceae using phylogenomics and spore coat protein-specific molecular markers
Source: Microbiol Spectr. 2026 Jun 15;14(7):e04185-25. doi: 10.1128/spectrum.04185-25 (PMC13339974; doi:10.1128/spectrum.04185-25)
Supplement: Supplemental material — Supplemental figure captions. [file spectrum.04185-25-s0003.docx]

****Figure S1.**** 16S rRNA phylogenetic tree constructed using the same 51 Peptostreptococcaceae strains as those employed for the phylogenomic tree based on concatenated sequences of genome-wide single‑copy orthologous proteins in Figure 2.

****Legend:**** Maximum likelihood tree inferred from the 16S rRNA gene sequences of 51 Peptostreptococcaceae strains (note that some strain accession numbers differ from those in Figure 2). Nodal values represent bootstrap support percentages obtained from 1,000 replicates. This figure is intended to enable direct comparison, at the same taxonomic scale, with the phylogeny derived from concatenated sequences of genome‑wide single‑copy orthologous proteins, and visually illustrates the limited resolution of the 16S rRNA marker at the species–genus level relative to phylogenomic (proteome‑based) phylogenetic analysis.

**Figure S2.** Full bootstrap support values for all nodes of the maximum likelihood phylogenetic tree corresponding to main Figure 5B

****Legend:****This tree was constructed based on the concatenated sequences of SpoIVA, YabG and CotE, with bootstrap values calculated from 1,000 replicates. The main Figure 5B only labels nodes with bootstrap support values ≥70%, while this supplementary figure provides the complete bootstrap values for all nodes in the tree.
